# S96. CARDIOVASCULAR DISEASE RISK IN PATIENTS WITH SCHIZOPHRENIA AND BIPOLAR DISORDER

**DOI:** 10.1093/schbul/sby018.883

**Published:** 2018-04-01

**Authors:** Linn Rødevand, Nils Eiel Steen, Daniel Quintana, Elina Johanna Reponen, Ragni Helene Mørch, Synve Hoffart Lunding, Trude Iversen, Trine V Lagerberg, Ingrid Melle, Ole A Andreassen

**Affiliations:** NORMENT & K.G. Jebsen Centre for Psychosis Research, Oslo University Hospital, University of Oslo

## Abstract

**Background:**

Patients with schizophrenia and bipolar disorder have markedly reduced life expectancy compared to the general population (15–20 years). A major contributor of excess death is cardiovascular disease (CVD) [1]. During the last decade, there have been several public health campaigns for health promotion and disease prevention, and tobacco legislation has become stricter. These strategies appear to have been effective in improving the health of the general Norwegian population [2]. It is unknown whether the elevated CVD risk in patients with schizophrenia and bipolar disorder has sustained in spite of these health promotion approaches. Here we investigate the development of CVD risk factors in a large representative sample of Norwegian patients with schizophrenia and bipolar disorder between 2002 and 2017. More specifically, we explored whether the CVD risk level was similar in a cohort from 2006 and a second cohort from 2017.

**Methods:**

Cross sectional analysis was performed among DSM-IV diagnosed patients included from 2002–2005 (cohort 1) and from 2006–2017 (cohort 2), respectively. Cohort 1 consisted of 161 patients with schizophrenia and 109 patients with bipolar disorder, and cohort 2 consisted of 623 patients with schizophrenia and 387 patients with bipolar disorder. Comparisons were made between cohorts regarding demographic variables, psychiatric symptoms, tobacco use, body mass index, waist circumference, total cholesterol, high-density lipoprotein (HDL) cholesterol, low-density lipoprotein (LDL) cholesterol, triglycerides, fasting glucose, systolic blood pressure, and diastolic blood pressure. ANCOVA was used for these analyses, with adjustments for age, duration of the disorder, and duration of psychopharmacological treatment.

**Results:**

Mean age was significantly higher for cohort 1 (35.1 years vs. 31.2 years, p < .001). There was no statistically significant difference in any of the other demographic variables or symptoms. Among patients with schizophrenia, there was no significant difference in the prevalence of CVD risk factors except from glucose being slightly increased in patients included in cohort 2 (p = .047). Among patients with bipolar disorder, there was a significant reduction in the level of total cholesterol, LDL, systolic, and diastolic blood pressure in cohort 2 (all p values < .01). These differences remained statistically significant after adjusting for age, duration of the disorder, and duration of psychopharmacological treatment.

**Discussion:**

Despite major advances in health promotion and disease prevention during the past decade, the level of CVD risk factors has remained high in patients with schizophrenia. While the level of some CVD risk factors improved in patients with bipolar disorder, they are still at increased risk of CVD. Thus, patients with severe mental disorders, especially schizophrenia, do not appear to have benefited from recent health promotion measures. Our findings also highlight the need for more effective interventions to reduce the risk of CVD in individuals with severe mental disorders, which may reduce the gap in life expectancy compared to the general population.

**References:**

1. Nordentoft M, Wahlbeck K, Hallgren J, et al. Excess mortality, causes of death and life expectancy in 270,770 patients with recent onset of mental disorders in Denmark, Finland and Sweden. PLoS One 2013;8(1):e55176. doi: 10.1371/journal.pone.0055176

2. Mannsverk J, Wilsgaard T, Mathiesen EB, et al. Trends in Modifiable Risk Factors Are Associated With Declining Incidence of Hospitalized and Nonhospitalized Acute Coronary Heart Disease in a Population. Circulation 2016;133(1):74–81. doi: 10.1161/circulationaha.115.016960

